# Quantitative Proteomics Analysis of Altered Protein Expression in the Placental Villous Tissue of Early Pregnancy Loss Using Isobaric Tandem Mass Tags

**DOI:** 10.1155/2014/647143

**Published:** 2014-03-13

**Authors:** Xiaobei Ni, Xin Li, Yueshuai Guo, Tao Zhou, Xuejiang Guo, Chun Zhao, Min Lin, Zuomin Zhou, Rong Shen, Xirong Guo, Xiufeng Ling, Ran Huo

**Affiliations:** ^1^State Key Laboratory of Reproductive Medicine, Nanjing Medical University, Nanjing 210029, China; ^2^Department of Histology and Embryology, Nanjing Medical University, Nanjing 210029, China; ^3^Nanjing Maternity and Child Health Hospital, Nanjing Medical University, Nanjing 210029, China

## Abstract

Many pregnant women suffer miscarriages during early gestation, but the description of these early pregnancy losses (EPL) can be somewhat confusing because of the complexities of early development. Thus, the identification of proteins with different expression profiles related to early pregnancy loss is essential for understanding the comprehensive pathophysiological mechanism. In this study, we report a gel-free tandem mass tags- (TMT-) labeling based proteomic analysis of five placental villous tissues from patients with early pregnancy loss and five from normal pregnant women. The application of this method resulted in the identification of 3423 proteins and 19647 peptides among the patient group and the matched normal control group. Qualitative and quantitative proteomic analysis revealed 51 proteins to be differentially abundant between the two groups (≥1.2-fold, Student's *t*-test, *P* < 0.05). To obtain an overview of the biological functions of the proteins whose expression levels altered significantly in EPL group, gene ontology analysis was performed. We also investigated the twelve proteins with a difference over 1.5-fold using pathways analysis. Our results demonstrate that the gel-free TMT-based proteomic approach allows the quantification of differences in protein expression levels, which is useful for obtaining molecular insights into early pregnancy loss.

## 1. Introduction

Many pregnant women suffer miscarriages during early gestation, and early pregnancy loss (EPL) has an adverse effect on the quality of life. The incidence of EPL ranges from 50% to 70% [1]. The epidemiological factors contributing to EPL are chromosomal defects of the conceptus, maternal age, endocrine diseases, anatomical abnormalities of the female genital tract, infections, immune factors, chemical agents, hereditary disorders, trauma, maternal diseases, psychological factors, and other such factors [1, 2]. Cytogenetic evaluation of sporadic spontaneous abortions has shown that 50–70% are chromosomally abnormal [3, 4]. The etiologies of EPLs are complex and some occur for unknown reasons. Therefore, the etiology of EPL remains to be further explored. The placenta is the organ that transports nutrients, respiratory gases, and wastes between the maternal and fetal systems. In the early embryonic development, the placental barrier facilitates the embryo growth in a low oxygen environment, effectively avoiding radical damage; once embryogenesis is complete, the maternal intervillous circulation becomes fully established, and the intraplacental oxygen concentration rises threefold and syncytiotrophoblastic oxidative damage becomes extensive and likely a major contributory factor to miscarriage [5]. The vascular development of the placenta is important for implantation [6]. It appears likely that apoptosis in the cytotrophoblast might be related to one aspect of the proliferation and degeneration of the trophoblast during early pregnancy [7]. There is speculation that an abnormal placenta leads to early abortion, but the unclear relationship between the placenta and early abortion remains requiring further elucidation.

Proteins that perform biological functions directly are rich in information that has been extremely valuable for the description of biological processes. The correlation between mRNA/DNA and protein levels is insufficient to predict protein expression levels [8]. Proteomics has many advantages compared with other technical means of analysis. Mass spectrometry (MS) allows the multivariate analysis of complex patterns of new biomarkers without knowledge of their individual identities and without having specific antibodies available [9]. A deeper knowledge of the human proteome could help fill the gap between genomes and phenotypes, transforming the way we develop diagnostics and therapeutics, and thereby enhancing overall biomedical research and future healthcare [10]. Recently, proteomics technologies have become sufficiently advanced enough that it is now realistic to measure clinical material to investigate pregnancy-related illnesses such as preeclampsia, Down syndrome pregnancies, and other such conditions [11–14]. The comparison of the proteome of interest between the healthy and disease state thus provides a fine-grained picture of the regulations involved [15]. In 2006, Liu et al. extracted protein from the placental villous tissue of two groups (six placental villous tissues with early pregnancy loss and six from normal pregnant women). They used the proteins from the placental villous tissue of the different groups as a source and 2D-gel-based proteomics as a discovery tool with the aim of discovering differentially expressed externalized proteins in EPL samples [16]. In their study, they identified several proteins associated with placentation and early development, granting new insight into the proteins involved in the pathophysiological mechanisms of early pregnancy loss. 2DE has been a mature technique for more than 25 years and was the first technique capable of supporting the concurrent quantitative analysis of large numbers of gene products. However, in many studies using this technology, the same proteins have largely been identified repeatedly, irrespective of the system studied, which suggests the limited dynamic range of 2DE-based proteomics [17, 18]. In addition, questions remain concerning the ability of the technique to characterize all of the elements of a proteome. Some studies indeed have revealed that typically only the most abundant proteins can be observed using the method [19, 20].

In this study, we aimed to (1) characterize the proteomes of placenta villous tissue using a gel-free proteomics approach, (2) apply high performance liquid chromatography (HPLC)-MS using an isobaric tandem mass tags (TMT) technology to identify differentially abundant proteins between the two groups, (3) bioinformatically analyze the differentially abundant proteins to determine the molecular function, biological process, and signaling pathways using gene ontology (GO) analysis and Pathway Studio software (v6.00), and (4) validate our results by western blot, immunohistochemistry, and pathway analysis of these differentially expressed proteins, which are useful for providing molecular insights into EPL.

## 2. Materials and Methods

### 2.1. Clinical Specimen Collection and Preparation

Tissue samples of five early pregnancy loss patients and five normal pregnant women were collected after obtaining informed consent and approval by the Human Studies Committee of Nanjing Maternity and Child Health Hospital (Nanjing, China). The pregnant women providing the samples had vaginal bleeding and/or lower abdominal pain for the first time in the previous few days (0–2 days). The diagnosis of EPL was based on the clinical history, clinical examination, and transvaginal ultrasound (TVU) results. In cases where pregnancy structures (a gestational sac without fetal heart rate) were identified by TVU, the final diagnosis of EPL was made. In control group, pregnancy structures are normal. Inclusion criteria were a gestational age at 7 to 8 weeks (based on the first day of the last menstrual period) and no history of recurrent spontaneous abortions, chromosomal abnormalities, endocrine diseases, anatomical abnormalities of genital tract, infections, immunologic diseases, trauma, internal diseases, hereditary disorders, maternal diseases, psychological factors, or any chemical agent intake before their elective terminations [16]. Experimental and control groups have similar physiological signs.

Placental villous tissue samples were acquired through the cervix during dilatation and aspiration according to strict clinical procedures. The tissue samples were washed in cold normal sterile phosphate buffered saline (PBS) to eliminate maternal blood, and deciduas were removed carefully under a microscope. In addition, each specimen was analyzed by hematoxylin-eosin staining to make sure there is no decidual contamination. Each sample was divided into three parts. One part was used as a cell culture for karyotype assays. Another part was fixed in 4% paraformaldehyde for 24 h at 4°C followed by paraffin embedding, and the third part was stored in liquid nitrogen until extraction. After evaluation via karyotype assays, five normal and five placental villous tissue early pregnancy loss samples, determined not to have embryonic chromosomal abnormalities, were selected for further study and defined as control (CON) samples and EPL samples, respectively.

### 2.2. Proteomics

#### 2.2.1. Protein Extraction and Digestion

A total of 300 *μ*g of each placental villous sample was homogenized and lysed using a protein extraction buffer consisting of 7 M urea, 2 M Thiourea, 65 mM DTT, and a 1% (v/v) protease inhibitor cocktail. The protein concentration was estimated by Bradford's method [21] using bovine serum albumin as the standard. Cysteine residues were reduced by incubating with DTT for 1 hour at 56°C followed by alkylation with IAA for 45 min at room temperature (RT) in the dark. The protein lysates were cleaned up by acetone precipitation and digested overnight at 37°C with trypsin in a 1 : 40 enzyme : protein ratio.

#### 2.2.2. TMT Labeling

The six-plex TMTs Label Reagents (Frankfurt am Main, Germany) were equilibrated to room temperature, and each aliquot was resuspended in 41 *μ*L of anhydrous acetonitrile. Samples were divided into two groups and respectively labeled as follows: Group 1: TMT-126, EPL-1; TMT-127, EPL-2; TMT-128, EPL-3; TMT-129, CON-1; TMT-130, CON-2, and TMT-131 labeled reference sample, which were pooled equally from all the ten samples. Group 2: TMT-126, CON-3; TMT-127, CON-4; TMT-128, CON-5; TMT-129, EPL-4; TMT-130, EPL-5 and TMT-131, pooled reference sample. Each experimental sample was compared to a pooled reference sample. After normalization, all samples were combined for comparison (5 abnormal samples versus 5 control samples) [22]. In addition, 42 *μ*L of the TMT Label Reagents was added to the 100 *μ*g of peptides dissolved in 200 mM triethylammonium bicarbonate (TEAB). After 60 min at RT, 8 *μ*L of hydroxylamine 5% (w : v) was added in each tube, and were incubated for 15 min. The aliquots were then combined, and the pooled sample was evaporated under vacuum.

#### 2.2.3. SCX Fractionation

The peptide mixture was resuspended in SCX chromatography Buffer A (10 mM NH_4_COOH, 5% ACN, pH 2.7) and loaded onto a cation ion exchange column (1 mm ID × 10 cm packed with Poros 10 S, DIONEX, Sunnyvale, CA) with the UltiMate 3000 HPLC system at a flow rate of 50 *μ*L/min. The following linear gradient was used: 0% to 56% B (800 mM NH_4_COOH, 5% ACN, pH 2.7) over 40 min, 56% to 100% B over 1 min, 100% B over 3 min, 100% to 0% B over 1 min, and 0% B for 20 min before the next run. The effluents were monitored at 214 nm based on the UV-light trace, and the fractions were collected every 2 min.

#### 2.2.4. LC-MS/MS

Twenty fractions were sequentially loaded onto a *μ*-precolumn cartridge (0.3 × 5 mm, 5 *μ*m, 100 Å; DIONEX, Sunnyvale, CA) at a flow rate of 0.3 *μ*L/min. The trap column effluent was then transferred to a reverse-phase microcapillary column (0.075 × 150 mm, Acclaim PepMap100 C18 column, 3 *μ*m, 100 Å; DIONEX, Sunnyvale, CA). The reverse-phase separation of peptides was performed using the following buffers: 2% ACN, 0.5% acetic acid (buffer A) and 80% ACN, 0.5% acetic acid (buffer B); a 219 min gradient (0% to 4% buffer B for 8 min, 4% to 9% buffer B for 3 min, 9% to 33% buffer B for 170 min, 33% to 50% buffer B for 10 min, 50% to 100% buffer B for 1 min, 100% buffer B for 8 min, 100% to 4% buffer B for 1 min, and 4% buffer B for 18 min) was used.

Peptides were analyzed using an LTQ Orbitrap Velos (ThermoFinnigan, San Jose, CA) by means of a data-dependent Top10-MS2/MS3 method [23]. For each cycle, one full MS scan of mass/charge radio (m/z) = 350 to 1800 was acquired in the orbitrap at a resolution of 30,000 or 60,000. Each full scan was followed by the selection of the 10 most intense ions for collision-induced dissociation (CID) and MS2 analysis in the linear ion trap for peptide identification. Each full scan was followed by the selection of the 10 most intense ions for collisioninduced dissociation (CID) and MS2 analysis in the linear ion trap for peptide identification and subsequent higher-energy collisional dissociation (HCD) and MS3 analysis in the Orbitrap for quantification of the TMT reporter ions [24].

#### 2.2.5. Protein Identification and Quantification

The resulting spectra were searched against the human IPI Protein Sequence Database (version: 3.83, 93, 289 sequences) [25] using MaxQuant (version: 1.2.2.5) software [26]. A common contaminants database was also included for quality control. Except for the TMT quantification labels, carbamidomethylation of cysteine was set as a fixed modification, and oxidized methionine was set as a variable modification. The initial mass tolerances for protein identification on the MS and MS/MS peaks were 20 ppm and 0.5 Da, respectively. Two missed cleavages were permitted, and full cleavage by trypsin was used. The false positive rates (FDRs) of the identified proteins and peptides were estimated by MaxQuant using a reverse strategy. A cutoff value of 1% was used for the identification of peptides and proteins. Protein quantification was calculated by combining MaxQuant identification results with a local modified Libra algorithm [27]. Proteins were considered differentially expressed when they displayed significant changes (more than 1.2-fold and Student's *t*-test *P*-value < 0.05) between the EPL and control groups.

### 2.3. Bioinformatics Analysis

For the convenience of gene annotation, corresponding Entrez gene IDs of the proteins were used for further bioinformatics analysis. To obtain an overview of the biological functions of the proteins whose expression levels altered significantly in EPL group, gene ontology analysis was performed using WebGestalt (Update 2013) [28]. To further explore the significance of the protein expressional alternation in the EPL group, we used Pathway Studio (v6.00) software (Ariadne Genomics, MD, USA) to search the regulated cellular processes of the proteins that displayed an over 1.5-fold difference between the EPL group and control group.

### 2.4. Validate the Proteomics Analysis

Western blot analysis of GSTM2 (glutathione S-transferase mu 2 (muscle)), BCS1L (BC1 (ubiquinol-cytochrome c reductase) synthesis-like), CUL7 (cullin 7), and immunohistochemistry analysis on proteins (GSTM2, BCS1L, FAM21 (family with sequence similarity 21), and CUL7) validated the proteomics analysis, they were randomly selected. Detailed steps are in Supplementary Material and method available online at http://dx.doi.org/10.1155/2014/647143.

## 3. Results

### 3.1. Protein Profiles of Placental Villous Tissue

We successfully identified proteins from placenta villous specimens of the EPL and control groups. In total, 3423 proteins were identified (Data S1) with high confidence (one or more unique peptides with an FDR less than 1%). The detailed information of the identified peptides is shown in Supplemental Data 2.

### 3.2. Identification of Differentially Expressed Proteins in Placental Villous Tissue from EPL Women by TMT Technology

Qualitative and quantitative proteomics analysis revealed 51 proteins as differentially abundant between the two groups (≥1.2-fold, Student's *t*-test, *P* < 0.05) (Data S3). GO analysis revealed both the molecular function and biological process of the differentially expressed proteins (Graph S1). In terms of molecular function, the most striking tendency is that there are a larger proportion of binding proteins (enzyme binding∖ubiquitin protein ligase binding∖small conjugating protein ligase binding molecule or a portion thereof). In terms of the biological processes, the top-ranked categories include organonitrogen compound metabolic process and intracellular transport. Among these proteins, 12 proteins were determined to be differentially abundant between the two groups with a fold change ≥1.5 (Student's *t*-test, *P* < 0.05) using this method. Five identified proteins (TBC1D13 (TBC1 domain family, member 13), LARP4B (La ribonucleoprotein domain family, member 4B), AHSG (alpha-2-HS-glycoprotein), P4HA2 (prolyl 4-hydroxylase, alpha polypeptide II), and BCS1L) were clearly upregulated, and 7 proteins (GSTM2, CUL7, NES (nestin), RASIP1 (Ras interacting protein 1), SLC30A2 (solute carrier family 30 (zinc transporter), member 2), PBXIP1 (pre-B-cell leukemia homeobox interacting protein 1), and FAM21) were downregulated in the EPL samples compared with the normal samples.

### 3.3. Western Blotting Analysis of Differentially Expressed Proteins

**Figure 1:**
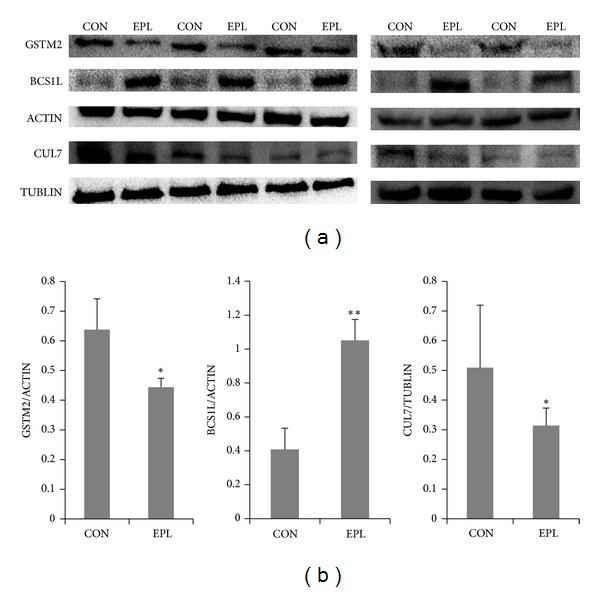
(a) Western blot analysis of GSTM2, BCS1L, and CUL7 of the control and EPL samples. (CON), control group; (EPL), EPL group; (b) the bar represents the relative gray values of the western blotting (*P < 0.05; **P < 0.01), Student's *t*-test, *P* value of GSTM2/ACTIN is 0.013700837; *P* value of BCS1L/ACTIN is 0.000039685; *P* value of CUL7/TUBLIN is 0.035421545.

To validate proteomics data, we performed western blot analysis on the same lysates of three proteins identified via MS, choosing to analyze proteins with different functions. The proteins confirmed by western blot were GSTM2, BCS1L, and CUL7; they were randomly selected. All of the proteins analyzed by western blot substantially verified the expressional alterations obtained by MS (Figure 1).

### 3.4. Detection of Differentially Expressed Proteins by Immunohistochemistry

**Figure 2:**
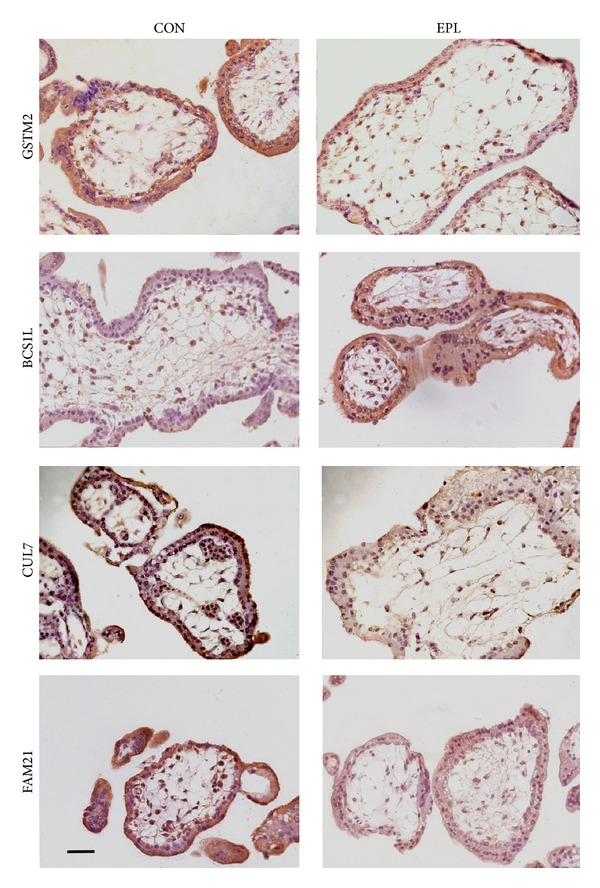
Immunohistochemistry analysis of GSTM2, BCS1L, CUL7, and FAM21. In these cases, cytoplasmic staining of the syncytiotrophoblastic and cytotrophoblastic cells was visualized in the placental villous tissues. The trends in the differential expression of the proteins were in accordance with the variation obtained from the quantitative proteomics analysis. (CON), control group; (EPL), EPL group; the bar represents 50 *μ*m.

Immunohistochemical studies were performed to examine the expression of some differentially expressed proteins, including GSTM2, BCS1L, CUL7, and FAM21; they were randomly selected. The results indicated the localization of these proteins. There are visualizable cytoplasmic staining of syncytiotrophoblastic and cytotrophoblastic cells in the placental villous tissues, and the signals in the tissues of control and EPL group also exhibited a similar expressional tendency as the proteomics data (representative results from each group were shown in Figure 2).

### 3.5. Pathway Analysis of the Differentially Expressed Proteins

**Figure 3:**
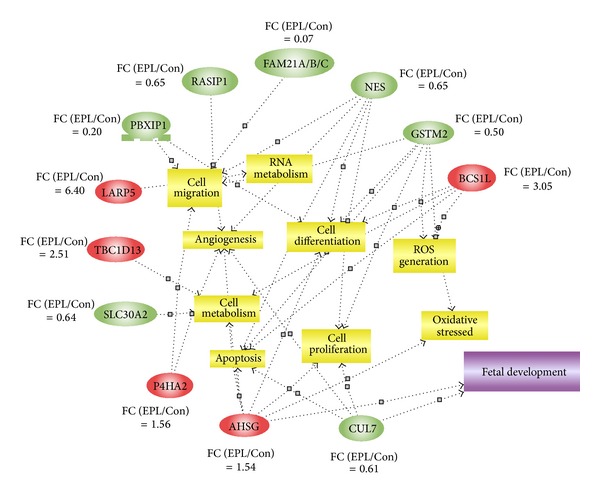
The cellular process annotation of 12 proteins that were differentially abundant between the two groups (≥1.5-fold). The cell cycle-related processes were generated by Pathway Studio software (v6.00). Proteins are shown as ovals, (five upregulated proteins were in red and 7 downregulated proteins were in green; the fold-change has been marked on the figures) and the regulated processes are represented by squares. The regulation events are indicated with arrows.

The simultaneous, cellular process annotation of 12 proteins that were differentially abundant between the two groups (≥1.5-fold, Student's *t*-test, *P* < 0.05) was generated by Pathway Studio software (v6.00) (Figure 3). The role of these proteins was characterized by cell regulations and processes such as cell migration, angiogenesis, oxidative stress, cell proliferation, apoptosis, and metabolism, among others, which indicates the risk factors in the EPL group.

## 4. Discussion and Conclusions

The placenta is a major contributor to pregnancy, and many pregnant women suffer miscarriage during early gestation, which has an adverse effect on the quality of life worldwide. Despite the considerable research efforts expended to understand the causes of these EPL, few significant advances have been made in recent decades because of the complexities of early development. Thus, the identification of proteins with different expression profiles related to EPL is essential to understand the comprehensive pathophysiological mechanism. In this study, we provide unique insights into differentially expressed proteins of placenta villous specimens from EPL subjects via HPLC-MS using TMT-labeling based technology; our data is useful for obtaining molecular insights into early pregnancy loss. We successfully identified 3423 proteins from placenta villous specimens in the EPL and control groups.

Qualitative and quantitative proteomics analysis revealed 51 proteins expressed differentially between the two groups (≥1.2-fold, Student's *t*-test, *P* < 0.05). Using WebGestalt, we performed BP and MF of the differentially abundant proteins. In terms of BP and MF, a majority of the proteins identified were transport/binding/metabolic components. This is because the placenta is an organ of plasticity that adapts to the needs of the fetus during gestation, which is obviously a multistep process [29]. Therefore, gene ontology analysis of differentially expressed proteins involves numerous factors that are involved in these processes, among them, 12 proteins with a difference over 1.5-fold (Student's *t*-test, *P* < 0.05). The role of these twelve proteins was further characterized by cell regulations and processes such as cell migration, angiogenesis, oxidative stress, cell proliferation, apoptosis, and metabolism among others. Western blot analysis of GSTM2, BCS1L, and CUL7 and immunohistochemistry of the proteins (GSTM2, BCS1L, FAM21, and CUL7) validated the proteomics analysis.

In embryonic development, trophoblast cells adhere and migrate down into the endometrium to form the hemochorial placenta. The early stages of placental development take place in a relatively hypoxic environment that favors cytotrophoblast proliferation rather than differentiation along the invasive pathway [30]. Among the differentially expressed proteins, NES, AHSG, BCSIL, GSTM2, and CUL7 are associated with cell proliferation and apoptosis. Some proteins, such as NES, P4HA2, PBXIP1, and GSTM2, may play a role in cell migration. NES, Cul7, RASIP1, P4HA2, and AHSG are associated with angiogenesis. NES encodes a member of the intermediate filament protein family, which were originally described in neural stem cells [31]. It is consistently expressed in adult angiogenic vasculature. NES expression has also been detected in capillaries of the corpus luteum, which replenishes itself by angiogenesis [32]. In the EPL group of our study, NES was decreased, which indicates that EPL may have a relationship with angiogenesis. In addition, vascular calcification is the most common type of extra-osseous calcification in end-stage renal disease (ESRD) [33]; AHSG has been demonstrated to exert a calcification inhibitory action both in vitro and in vivo [34, 35]. AHSG is increased in EPL, which may reflect vascular dysfunction in EPL.

The glutathione transferases (GSTs) are a complex family of enzymes involved in detoxification of a wide range of harmful chemicals, including environmental pollutants, carcinogens, mutagens, and toxic products such as lipid hydroperoxides generated during oxidative stress [36]. GSTM2 encodes a glutathione S-transferase. Important clues about oxygen's effects on the placenta have come from several lines of evidence that suggest that the early stages of placental (and embryonic) development take place in an environment that is hypoxic relative to the uterus [30]. As trophoblast invasion of the uterus proceeds, the placental cells encounter increasingly higher oxygen levels [37]. The syncytiotrophoblastic layer of the early placenta is exquisitely sensitive to rapidly rising oxygen tensions in vitro and undergoes selective degeneration [38]. Enzyme manganese superoxide dismutase (MnSOD) catalyzes the dismutation of O_2_^•−^ to hydrogen peroxide, which is, in turn, converted to oxygen and water by the enzyme catalase [39]. In our study, GSTM2 expression was low in the EPL groups, suggesting that GSTM2 is important in the maintenance of early pregnancy by preventing the oxidative stress, which is in general agreement with the findings of Liu et al. [16] who reported a decrease in the SOD level in the placental villi of EPL samples.

In addition, the mitochondrial respiratory chain is one of the most prolific producers of superoxide. It has been estimated that almost 1-2% of all electrons passing through the respiratory chain end up as superoxide ions [40]. The production of superoxide during hypoxia and ischemia in cardiovascular tissue can be very damaging [41]. BCS1L encodes a member of the AAA family of ATPases that is necessary for the assembly of complex III in the mitochondria [42]. The AAA-family ATPases mediate the folding, unfolding, assembly, and degradation of proteins [43, 44]. In Björnstad syndrome, BCS1L mutations cause complex III deficiency and GRACILE syndrome, which in neonates are lethal conditions that have multisystem and neurologic manifestations typifying severe mitochondrial disorders [45, 46]. BCS1L mutations alter assembly of the mitochondrial respirasome, reduce the activity of the electron transport chain, and increase the production of reactive oxygen species [42]. BCS1L expression was high in the EPL group, which may reflect the greater production of superoxide and may partly explain the malfunction of GSTM2 in EPL.

CUL7 is a large polypeptide containing a cullin domain, which is present in the anaphase-promoting complex [47]. CUL7 belongs to the cullin family. Cullins are proteins involved in ubiquitination through their participation in multisubunit ubiquitin ligase complexes [48]. Ubiquitination is a critical process in all eukaryotic organisms. It is involved in several essential functions, from the regulation of protein levels to roles in cellular signaling, DNA repair, endocytosis, and gene expression regulation [49, 50]. Researchers demonstrated that CUL7 is highly expressed in first trimester invasive human placental villi and a key inducer of the epithelial-mesenchymal transition (EMT) of trophoblast lineages [51]. CUL7 appears to be an important regulator of placental development. CUL7 knock-out in mice results in small and abnormal placentas [52]. In this study, CUL7 was decreased in EPL samples compared with normal pregnancy samples. EPL may be associated with ubiquitination and trophoblast migration/invasion. Our studies suggest a possible pathological mechanism for the CUL7-linked pathway in EPL for the first time.

In comparing our results with the published reports, we also observed differences that may be due to the different technical methods, but there are still commonalities identified in the functions of these proteins. Therefore, the use of different methods to elucidate the mechanism of the pathogenesis of EPL is necessary. We conclude that these globally profiled and differentially expressed proteins of placenta villous specimens from EPL women are helpful in obtaining molecular insights into EPL.

## Supplementary Material

Supplemental Docx1 provided the materials and methods such as reagents, western blot analysis and immunohistochemistry. Supplemental Data 1 gave the identified proteins information.
Supplemental Data 2 gave the identified peptide information. Supplemental Data 3 listed the human placental villous tissue proteins that were differentially abundant between the two groups identified in Human placental villous (≧1.2-fold). Supplemental Graph 1 showed the GO analysis in both the molecular function and biological process of the differentially expressed proteins.
