# Supplementary material for: Quantitative Proteomics Analysis of Altered Protein Expression in the Placental Villous Tissue of Early Pregnancy Loss Using Isobaric Tandem Mass Tags
Source: Biomed Res Int. 2014 Mar 13;2014:647143. doi: 10.1155/2014/647143 (PMC3971554; doi:10.1155/2014/647143)
Supplement: Supplementary file 1 — Supplemental Docx1 provided the materials and methods such as reagents, western blot analysis and immunohistochemistry. Supplemental Data 1 gave the identified proteins information. Supplemental Data 2 gave the identified peptide information. Supplemental Data 3 listed the human placental villous tissue proteins that were differentially abundant between the two groups identified in Human placental villous (≧1.2-fold). Supplemental Graph 1 showed the GO analysis in both the molecular function and biological process of the differentially expressed proteins. [file 647143.f1.zip › Supplementray Materials/Supplemental1.docx.docx]

**supplemental materials and methods**

**1.1 Reagents**

Hematoxylin (Sigma Chemical, St. Louis, MO, USA), eosin (Sigma Chemical), urea (GE Healthcare, Uppsala, Sweden), thiourea (Sigma Chemical), dithiothreitol (DTT) (GE Healthcare), protease inhibitor cocktail (Pierce Biotechnology, Rockford, IL), iodoacetamide (IAA, GE Healthcare), trypsin (Promega, Madison, WI, USA), trifluoroacetic acid (TFA) (Sigma Chemical), ammonium formate (Sigma Chemical), acetonitrile (CAN, Sigma Chemical), acetic acid (Sigma Chemical), ammonium bicarbonate (NH_4_HCO_3_, Sigma Chemical) were acquired and use in the experiments. In addition, 3-[(3-cholamidopropyl)-dimethylammonio]-1-propane sulfonate (CHAPS)， tris-(hydroxymethyl)-aminomethane (Tris), acrylamide, methylene bis-acrylamide, sodium dodecyl sulphate (SDS), ammonium persulfate (APS), N,N,N′,N′-tetramethylethylenediamine (TEMED), and enhanced chemiluminescence reagents were purchased from Amersham Bioscience (Uppsala, Sweden). The 6-plex Tandem Mass Tags were provided by Thermo scientific. Antibody to human BCS1-like (Bcs1L) (Proteintech), glutathione S-transferase mu2 (Gstm2) (Abcam), Anti-WASH complex subunit Fam21 (Fam21) (Millipore) and ubiquitin ligase cullin 7 (CUL7) (Bethyl Laboratories), HRP-conjugated secondary antibody (Beijing ZhongShan Biotechnology, China) were also used. All other chemicals were acquired from Nanjing Chemical Company (Nanjing, China).

**1.2 Western Blot Analysis**

The frozen tissues were homogenized with an ULTRA TURRAX homogenizer (Ika, Petaling Jaya, Malaysia) in lysis buffer that contained 7 M urea, 2 M thiourea, 4% (w/v) CHAPS, 2% (w/v) DTT, a 1% (v/w) Protease Inhibitor Cocktail (Pierce Biotechnology, Rockford, IL, USA) at 11,000 IU/min on ice (10 bursts of 10 s, each interspersed with short pauses). The suspensions were kept shaking at 4°C for 1 h, and insoluble molecules were removed by centrifugation at 40000 × g at 4°C for 1 h. The protein concentration in each sample was determined by the Bradford method using BSA as the standard. Samples that contained 50-100 μg of protein from the two groups were electrophoresed on a 12% SDS polyacrylamide gel and transferred to a nitrocellulose membrane (GE Healthcare, San Francisco, CA, USA). The membranes were blocked in Tris-buffered saline (TBS) that contained 5% non-fat milk powder for 1 h, and incubated in anti-Bcs1l (1:100, Proteintech), anti-Gstm2 (1:500, Abcam), anti-cul7 (1:500, Bethyl Laboratories), and anti-β-actin/β-Tublin (1:1000, Abcam), diluted in TBS/5% non-fat milk powder overnight. β-Actin/β-Tublin was used as a loading control. The membranes were washed 3 times (10 min each) with TBS and incubated for 1 h with horseradish peroxidase (HRP)-conjugated goat anti-rabbit IgG (1:1000, Beijing ZhongShan Biotechnology, Beijing, China). The specific proteins were detected using an ECL kit and AlphaImager (FluorChem5500; Alpha Innotech).

**1.3 Immunohistochemistry of placental villous tissue**

Sections from fixed samples were analyzed by immunohistochemistry. Briefly, tissues were embedded in paraffin, separated into 5μm sections. The sections were dewaxed and rehydrated through descending grades of alcohol to distilled water and subjected to a microwave antigen retrieval technique, and the endogenous peroxidase activity was quenched. After that, the sections were blocked using a blocking serum and then incubated overnight at 4°C with anti-Bcs1l (1:100, Proteintech), anti-Gstm2 (1:500, Abcam), anti-WASH complex subunit Fam21 (1:1000, Millipore), anti-cul7 (1:500, Bethyl Laboratories). The next day, the sections were incubated with HRP-conjugated secondary antibody. Immunoreactive sites were visualized as brown.
